# Efficacy of Feed Additives on Immune Modulation and Disease Resistance in Tilapia in Coinfection Model with Tilapia Lake Virus and *Aeromonas hydrophila*

**DOI:** 10.3390/biology13110938

**Published:** 2024-11-16

**Authors:** Aslah Mohamad, Jidapa Yamkasem, Suwimon Paimeeka, Matepiya Khemthong, Tuchakorn Lertwanakarn, Piyathip Setthawong, Waldo G. Nuez-Ortin, Maria Mercè Isern Subich, Win Surachetpong

**Affiliations:** 1 Department of Veterinary Microbiology and Immunology, Faculty of Veterinary Medicine, Kasetsart University, Bangkok 10900, Thailand; aslahumt@gmail.com (A.M.); jidapa.yam@ku.th (J.Y.); suwimon.pai@ku.th (S.P.); matepiya.kh@ku.th (M.K.); 2 Department of Physiology, Faculty of Veterinary Medicine, Kasetsart University, Bangkok 10900, Thailand; tuchakorn.l@ku.th (T.L.); piyathip.s@ku.th (P.S.); 3 ADISSEO France S.A.S., Immeuble Antony Parc 2 10, Place du Général de Gaulle, 92160 Antony, France; waldo.nuezortin@adisseo.com (W.G.N.-O.); mariamerce.isern@adisseo.com (M.M.I.S.); 4 Laboratory of Biotechnology, Chulabhorn Research Institute, Bangkok 10210, Thailand

**Keywords:** feed additives, TiLV, *Aeromonas hydrophila*, coinfection, aquaculture

## Abstract

Coinfections by multiple pathogens, including viruses and bacteria, have severely impacted tilapia aquaculture globally. This study evaluated the impacts of dietary supplementation on red hybrid tilapia (*Oreochromis* spp.) coinfected with Tilapia lake virus (TiLV) and *Aeromonas hydrophila*. Fish were divided into three groups: a control group on a normal diet, and two experimental groups received diets supplemented with strategy A, an organic acid blend combined with a lyso-phospholipid-based digestive enhancer, and strategy B, an organic acid blend combined with natural immunostimulants and nutrients. Following exposure to both pathogens, the fish supplemented with strategies A and B showed lower cumulative mortality rates of 50.0% and 41.7%, respectively, compared to 76.3% in the control group. Notably, fish fed with strategy B-supplemented diet displayed a stronger immune response, with a lower expression of *il-8*, *mx*, and *rsad2*, and showed less pathological changes in the liver, spleen, and intestines, suggesting enhanced resistance to coinfection. In contrast, fish receiving strategy A did not exhibit significant changes in the immune-related gene expression or pathogen load, but demonstrate less pathological alterations, indicating intestinal protection. These findings highlight the potential of feed additives, particularly strategy B, to reduce the impact of virus-bacterial coinfections and improve outcomes in tilapia farming.

## Simple Summary

In this study, we explored how two different dietary supplemental strategies can help red hybrid tilapia (*Oreochromis* spp.) combat infections caused by Tilapia lake virus (TiLV) and *Aeromonas hydrophila*. We found that these feed supplements boost fish’s immune system and improve their survival against these pathogens. Notably, strategy B was particularly effective, leading to lower mortality compared to those on a regular diet. Our results suggest that these dietary strategies could provide valuable support for tilapia farmers, allowing them to protect their fish from harmful diseases and ensure healthier stocks.

## 1. Introduction

Tilapia (*Oreochromis* spp.) is an important source of dietary protein and contributes to food security worldwide. However, the sustainability of tilapia aquaculture is under threat due to the rise in infectious diseases caused by viruses, bacteria, and parasites [1]. Notably, coinfections, which involve simultaneous or sequential attacks by multiple pathogens, pose challenges to the development of treatments and the identification of the primary pathogen response to reduce fish morbidity and mortality [1,2,3]. Various examples of coinfections reported in tilapia include Tilapia lake virus (TiLV), Tilapia parvovirus, *Aeromonas* spp., infectious spleen and kidney necrosis virus and *Streptococcus agalactiae*, and infections by iridoviruses, *Flavobacterium columnare*, and *A. veronii* [3,4,5,6]. These coinfections can synergistically make fish more susceptible to other pathogens, worsen clinical signs, increase mortality rates and consequently lead to significant economic losses in tilapia aquaculture [2].

TiLV and Aeromonas have emerged as significant causes of tilapia mortality in recent years. These pathogens are frequently found coinfecting tilapia in field conditions [3,5,7] and have been shown to cause severe infections [5]. Specifically, a study reported a 70% mortality in red tilapia (*Oreochromis* sp.) due to coinfection with TiLV and bacteria. In the affected fish, *Aeromonas hydrophila* was detected in 100% of cases, while *S. agalactiae* was found in 50% of the affected fish [5]. Moreover, the coinfected fish showed higher mortality rates compared to those infected with either pathogen alone. These findings emphasise the importance of addressing coinfections in TiLV outbreaks and highlight the need for comprehensive strategies to mitigate their impact. To minimise mortality caused by coinfections, strategies such as vaccinations, antibiotics and the application of bacteriophages may be implemented at fish farms [8,9]. Feed additives and probiotics have also gained attention for their capacity to reduce mortality and promote the health of fish. In tilapia, feed additives, such as phytogenic essential oils, acidifiers, organic acids, algae, and cyanobacteria, have shown promise in reducing mortality associated with *A. hydrophila* infections [10,11]. Similarly, the use of *Bacillus* spp. probiotics and feed additives containing *Saccharomyces cerevisiae* has been studied and shown to provide some protection against a single infection by TiLV or *A. hydrophila* through enhanced immune function in red hybrid tilapia [11]. However, the potential use of these feed additives against coinfections in tilapia has not been reported to date. In this study, we evaluated the efficacy of commercially available feed additives in reducing the impact of coinfection with TiLV and *A. hydrophila* in red hybrid tilapia by examining the cumulative mortality and immune responses. Our findings could provide valuable information for preventive strategies that will help manage these coinfections.

## 2. Materials and Methods

### 2.1. Experimental Fish

A total of 530 red hybrid tilapia with an average body weight of 7.0 ± 0.5 g were obtained from a hatchery in Petchaburi province and subsequently housed at the animal research facility of the Faculty of Veterinary Medicine, Kasetsart University, Bangkok province, Thailand. Upon arrival, the fish underwent a two-week acclimation period in a 400 L tank, and the water quality parameters were maintained as follows: dissolved oxygen (DO) at 5.68 ± 0.32 mg/L, ammonia (NH_3_) at 0.18 ± 0.05 mg/L, nitrite (NO_2_^−^) at 0.10 ± 0.05 mg/L, and pH at 7.20 ± 0.35. The water temperature was controlled at 28 °C ± 1 °C with a photoperiod of 12 h of light and 12 h of darkness. The fish were fed twice daily with commercial feed at a rate of 3% of total body weight, and their behaviour was observed daily for any abnormalities. Ten fish were randomly selected for screening for external parasites and bacterial and TiLV infection. All the procedures in the study were approved by the Kasetsart University Animal Use Committee (protocol number ACKU63-VET-011).

### 2.2. Preparation of Virus and Bacteria for the Challenge Study

The TiLV strain VETKU-TV08, which was isolated from moribund red hybrid tilapia in Ang Thong province, Thailand in 2019, was propagated in E-11 cells, which are clones of the SSN-1 cell line obtained from the European Collection of Authenticated Cell Cultures. The E-11 cells were cultured in Leibovitz’s L-15 medium supplemented with 5% foetal bovine serum and cultured at 25 °C until 80–90% confluence was reached. The infected cells were then passed into the confluent E-11 cells, and the subsequent cytopathic effect (CPE) was observed daily. Between 5 and 7 days post-virus inoculation, when an 80% CPE was visible, the viruses were harvested from the E-11 cells by centrifugation at 3000× *g* for 10 min at 4 °C. The supernatant containing the harvested viruses was stored at −80 °C until the challenge experiment. The TiLV titre (TCID_50_/mL) was determined by following the standard serial dilution protocol [12].

The bacterial challenge study was prepared from a suspension of the *A. hydrophila* isolates, KU-AH62. Briefly, the bacterial isolates were streaked onto Tryptic soy agar (TSA) (Difco™, Sparks, MD, USA) and incubated overnight at 30 °C. The next day, a full loop of *A. hydrophila* colonies was transferred to Tryptic soy broth (Difco™, Franklin Lakes, NJ, USA) and incubated overnight at 30 °C in an incubator shaker at 225 rpm. The bacterial concentration was determined using a standard plate count technique [13]. The bacterial suspension was then centrifuged at 3000× *g*, and the supernatant was discarded. The pellet was resuspended in a 0.85% NaCl solution and diluted to achieve a final concentration of 10^8^ CFU/mL.

### 2.3. Diet, Feed Additives and Experimental Design

**Figure 1:**
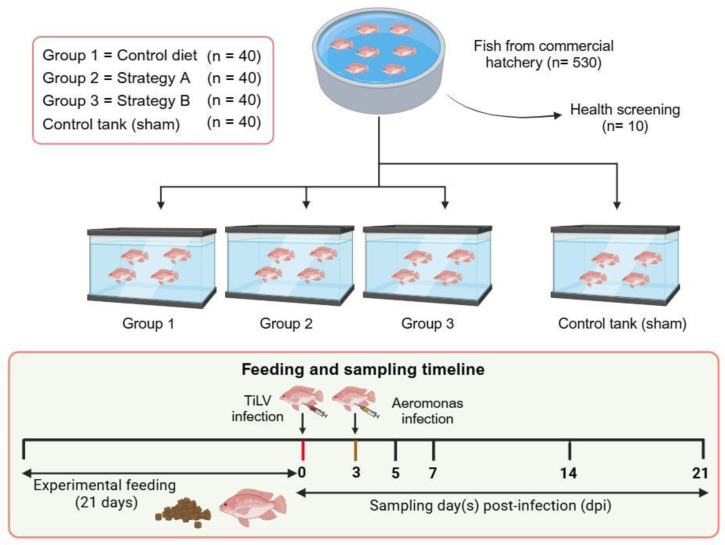
Experimental design of the study. The fish were fed a control diet only or feed supplemented with additives using strategy A or B for 21 days. Groups 1 to 3 had four replicates and one tank dedicated as the control tank (sham) group. Fish were injected intraperitoneally with TiLV at 10^5^ TCID_50_/mL on day 0 and *A. hydrophila* at 10^8^ CFU/mL on day 3.

A schematic of the experimental design of the study is presented in Figure 1. A total of 480 red hybrid tilapia were divided equally into three groups, with 40 fish per tank (150 L) in four replicates. Group 1 comprised positive control fish that received a standard diet, while the fish in Groups 2 and 3 were supplemented with additives using strategy A, 0.3% *w*/*w* of organic acid blend (Bacti-Nil^®^ Aqua Adisseo, Antony, France) combined with 0.1% *w*/*w* of a lyso-phospholipid-based digestive enhancer (Aqualyso^®^, Adisseo, Antony, France) and strategy B, a 0.3% *w*/*w* of organic acids blend combined with 0.1% w/w of a blend of natural immunostimulants and nutrients (Aquastim^®,^ Adisseo, Antony, France), respectively. The feed used in this study contained 32.9% protein and other components (Supplementary Table S1), with each feed additive (Strategy A and B) incorporated prior to the pelleting process.

After 21 days of feeding with the respective diets, the fish from each group were first challenged with 50 µL of TiLV at 10^5^ TCID_50_/mL intraperitoneally at 0 days post-infection (dpi) and subsequently with 100 µL of *A. hydrophila* at 10^8^ CFU/mL intraperitoneally at 3 dpi. Additionally, a negative (sham) control (*n* = 40) that received a control diet with an intraperitoneal injection of the L-15 medium collected from uninfected E-11 cells and 0.85% NaCl was conducted in parallel. The fish were fed twice daily at a rate of 3% of their total body weight. Tissue samples (liver, spleen, and anterior kidney) were collected at 0, 3, 5, 7, and 14 dpi from five fish per group. Three tanks were dedicated to record cumulative mortality, with an additional tank designated for sample collection. The samples were processed for bacterial detection, viral quantification, immune gene expression, and histopathological studies. The water quality parameters, including temperature, DO, NH_3_, NO_2_^−^, and pH, were examined daily. At 21 dpi, all the fish were euthanised using an overdose of eugenol solution at a concentration of 30 mL/L.

### 2.4. Detection of A. hydrophila in Infected Fish

The anterior kidney samples from three fish (*n* = 3) in each treatment and control group at each time point (0, 3, 5, 7, and 14 dpi) were collected to detect *A. hydrophila*. The samples were individually streaked onto TSA plates (Difco™, Sparks, MD, USA) and incubated overnight at 30 °C. Following incubation, the dominant uniform isolates were purified through successive streaking on TSA plates.

The purified isolates were then subjected to DNA extraction using the GF-1 Bacterial DNA Extraction Kit (Vivantis, Shah Alam, Selangor, Malaysia) according to the manufacturer’s protocol. The polymerase chain reaction (PCR) amplification of the gyrase B subunit (*GyrB*) gene specific for *Aeromonas* spp. was performed using a method described elsewhere [14]. The PCR was performed in a 20 μL reaction volume containing 1 × Taq polymerase buffer, 2 μL of MgCl_2_ (25 mM), 0.2 mM of dNTP mix, 0.125 μM of each primer, 0.25 μL of 5 U/μL Taq DNA polymerase, and 200 ng of DNA. The forward and reverse primer sequences were IAF: 5′-CTGAACCAGAACAAGACCCCG-3′ and IAR: 5′-ATGTTGTTGGTGAAGCAGTA-3′. The PCR was performed in a T100 thermal cycler (BioRad, Hercules, CA, USA) with the following cycle conditions: initial denaturation at 95 °C for 2 min, followed by 40 cycles of denaturation at 94 °C for 10 s, annealing at 55 °C for 10 s, and an extension at 72 °C for 10 s. The PCR products were separated on 2% (*w*/*v*) agarose gel electrophoresis and visualised using the ChemiDoc Imaging System (BioRad, Hercules, CA, USA).

### 2.5. Total RNA Extraction and cDNA Synthesis

The total RNA was extracted from the liver, spleen, and anterior kidney tissues collected at the designated experimental time points using Trizol^®^ (Invitrogen, Carlsbad, CA, USA) in line with the manufacturer’s instructions. The quality and quantity of the extracted RNA were assessed using a NanoDrop™ Spectrophotometer (Thermo Fisher Scientific, Waltham, MA, USA). Complementary DNA (cDNA) was synthesised from 1 µg of total RNA using a ReverTra Ace^®^ kit (Toyobo, Osaka, Japan) as per the manufacturer’s instructions. The cDNA was diluted with diethylpyrocarbonate water and stored at −80 °C for further analysis.

### 2.6. Quantitative PCR

The quantification of the TiLV genomic RNA in the liver, spleen, and anterior kidney samples and the expression levels of the immune-related genes (*il-8*, *ifn-γ*, *mx*, and *rsad2*) in the anterior kidney were analysed using a quantitative PCR (qPCR) assay in accordance with the method described previously [15]. Each reaction contained 5 µL of iTaq™ universal SYBR Green Supermix (BioRad, Hercules, CA, USA), 0.3 µM of both the forward and reverse primers for each gene (Table 1), 100 ng of cDNA template, and molecular-grade distilled water to adjust the total volume to 10 µL. The qPCR cycle condition consisted of an initial phase at 95 °C for 3 min, followed by 40 cycles at 95 °C for 10 s and at 60 °C for 30 s using the CFX96™ thermocycler (BioRad, Hercules, CA, USA). All the samples were processed in duplicate, and the average threshold cycle (Ct) values were retrieved to calculate the relative expression values. The TiLV concentration was determined by extrapolating the obtained quantification cycle on a standard curve derived from 10-fold serial dilutions of a plasmid containing TiLV segment 3 [15]. The relative expression level for each immune-related gene and viral concentration was determined by assessing the Ct differences between the reference gene (*β-actin*) in each sample (ΔCt), followed by the baseline level of the fish before infection (ΔΔCt) and employing the formula 2^−ΔΔCt^ [16].

### 2.7. Histopathology

The internal organs (liver, spleen, and intestines) were collected from five fish from the treatment and control groups at 0, 3, 5, 7, and 14 dpi. The tissue samples were fixed in 10% neutral buffered formalin, embedded in paraffin wax, sectioned to a 4 µM thickness, and stained with haematoxylin and eosin in line with the standard histopathological protocols. The slides were scanned and visualised using the VS120^®^ virtual microscopy slide scanning system (Olympus, Tokyo, Japan) and evaluated with the Olympus OlyVIA 3.1 programme (Olympus, Tokyo, Japan).

### 2.8. Statistical Analysis

The relative percent survival of both the control and treatment groups was determined and analysed using Kaplan–Meier analysis followed by a log-rank (Mantel–Cox) test. The viral concentrations and gene expression data from all samples were subjected to the Shapiro–Wilk test, and all data followed a normal Gaussian distribution. The comparisons of daily mortality, TiLV RNA, and gene levels between the additive and control diet groups at different time points were assessed by two-way ANOVA, followed by Tukey’s test using Prism software 8 (GraphPad Prism, San Diego, CA, USA). The data were presented as the mean ± standard deviation (S.D.). A *p*-value less than 0.05 was considered statistically significant.

## 3. Results

### 3.1. Influence of Additive Supplementation on Clinical Signs, Cumulative Mortality and Pathological Changes

**Figure 2:**
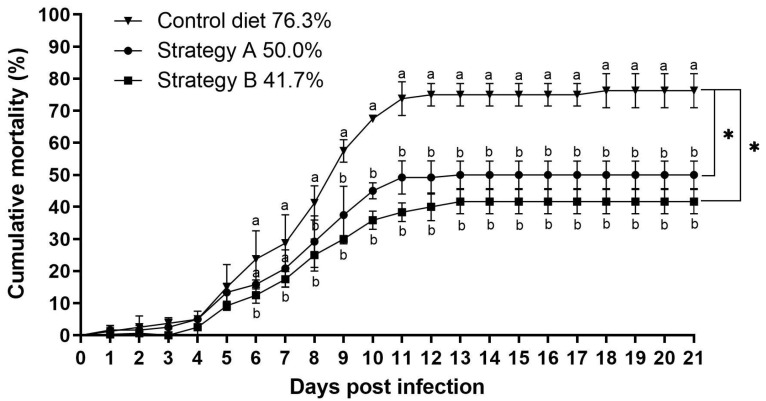
Comparison of the cumulative mortality (%) of red hybrid tilapia coinfected with Tilapia lake virus (TiLV) and *Aeromonas hydrophila* from the control group and the groups that received feed additives with strategy A or B. The asterisks (*) indicate significant differences in cumulative mortality (*p* < 0.05) compared to the control group.

**Figure 3:**
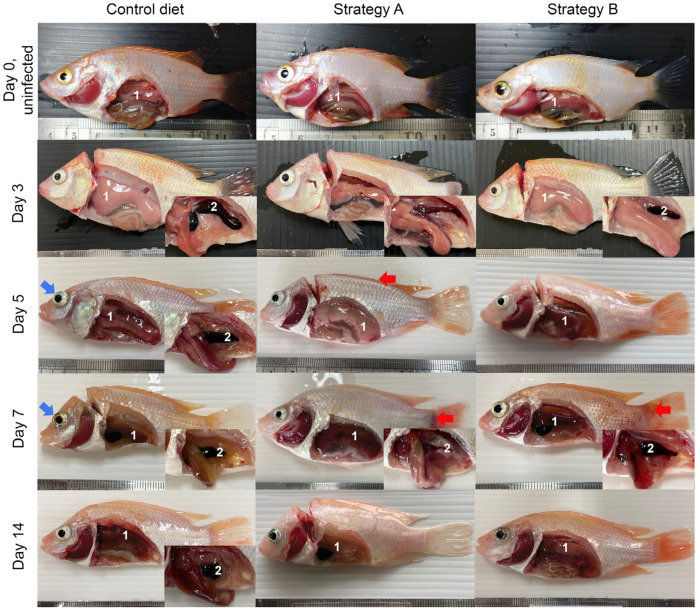
The external appearances and lesions of internal organs (1-liver; 2-spleen) of the fish in the control group and those that received feed additives with strategy A or B. During the challenge study (days 0 to 14), the infected fish showed haemorrhaging and congestion on the dorsal skin and fin base (indicated by red arrows) and exophthalmia (indicated by blue arrows). The common internal lesions had ascitic fluid, and a pale, yellow liver and an enlarged spleen (inset) were evident.

After being challenged with TiLV and *A. hydrophila*, all the fish showed clinical signs of reduced appetite, lethargy, and atypical swimming behaviours between 3 and 15 dpi. Indeed, the fish mortality started at 4 dpi and increased rapidly across all the groups, with cumulative mortality ranging from 41.7% to 76.3% (Figure 2). Notably, the highest cumulative mortality rate was observed in the control group. In contrast, the fish supplemented with either strategy A or B exhibited significantly lower mortality rates of 50.0% and 41.7%, respectively (*p* < 0.05). The gross pathology examination revealed external abnormalities, such as skin paleness and haemorrhages, abdominal swelling, fin erosion, and exophthalmos, in all the challenged fish. The internal pathological features included the presence of a yellowish protein-rich ascitic fluid, a pale and yellow liver, an enlarged spleen and anterior kidney, and oedematous intestines (Figure 3). No mortality or gross pathology was observed in the sham control fish.

### 3.2. Effects of Feed Additives on the Replication of TiLV and A. hydrophila

**Figure 4:**
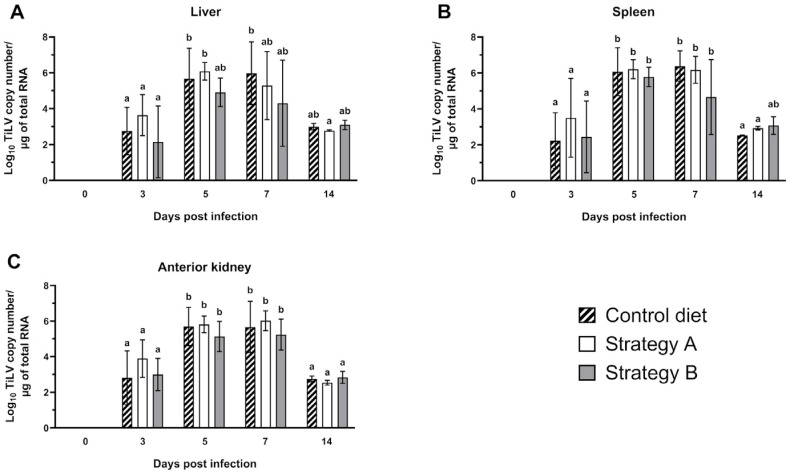
The levels of Tilapia lake virus (TiLV) RNA in the (**A**) liver, (**B**) spleen, and (**C**) anterior kidney of the fish fed the control diet or the feed supplemented with additives using strategy A or B at 0, 3, 5, 7, and 14 days post-infection. The data from five fish are presented as the mean ± standard deviation. Different alphabets indicate statistical differences (*p* < 0.05) between groups according to Tukey’s multiple comparison tests.

We compared the levels of TiLV RNA in various organs, including the liver, spleen, and anterior kidney, of the infected fish that received either a control diet or a diet supplemented with additives using strategy A or B (Figure 4). Notably, TiLV RNA was detected in the internal organs of all the challenged fish at 3 dpi, with levels ranging from log 2.15 ± 2.00 to log 3.64 ± 1.15 copies/µg of total RNA in the liver; log 2.23 ± 1.56 to log 3.50 ± 2.19 copies/µg of total RNA in the spleen; and log 2.81 ± 1.51 to log 3.89 ± 1.06 copies/µg of total RNA in the anterior kidney. The viral load increased remarkably in all organs, with peaks evident between 5 and 7 dpi and TiLV log concentrations ranging from log 4.30 ± 2.40 to log 6.08 ± 0.49 copies/µg of total RNA in the liver; log 4.66 ± 2.09 to log 6.38 ± 0.84 copies/µg of total RNA in the spleen; and log 5.13 ± 0.85 to log 6.02 ± 0.55 copies/µg of total RNA in the anterior kidney across all the groups. By 14 dpi, the viral load decreased in all the infected fish and returned to levels similar to those observed in the early stage of infection (Figure 4). Nevertheless, there were no statistically significant differences in the TiLV load in the liver, spleen, and anterior kidney of the groups at any time point (*p* > 0.05). TiLV was not detected in the fish at 0 dpi or in the sham-challenged group throughout the studied period. 

Meanwhile, we conducted bacterial isolation and PCR to detect *A. hydrophila* infection in the anterior kidney of the infected fish at 0, 3, 5, 7, and 14 dpi (Table 2). Our results showed bacteria in all the infected fish at 5 and 7 dpi (Supplementary Figure S1). However, no bacterial growth was detected in any of the groups at 0, 3, and 14 dpi. Additionally, there were no statistically significant differences (*p* > 0.05) in *A. hydrophila* detection between the infected control fish and those supplemented with additives using strategy A or B throughout the sampling period.

### 3.3. Impact of Feed Additives on the Expression of Immune-Related Genes

**Figure 5:**
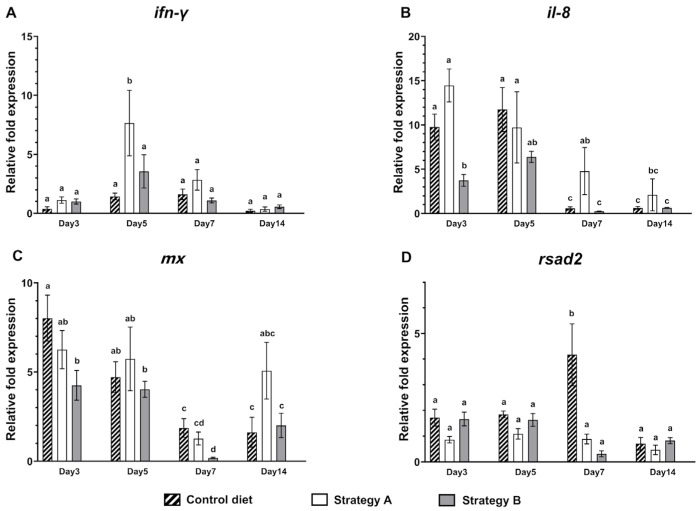
Expression of the (**A**) *ifn-γ*, (**B**) *il-8*, (**C**) *mx*, and (**D**) *rsad2* genes from the anterior kidneys of the challenged fish that were fed the control and additive-supplemented diets, strategies A and B. The samples were collected at day 0 and 3, 5, 7 and 14 days post-infection following the Tilapia lake virus and bacterial challenge (*n* = 5). The relative fold expression of genes at each time point following infection was normalised to day 0 and is presented as the mean ± standard deviation. Significant differences (*p* < 0.05) between groups from Tukey’s multiple comparison tests were denoted by different letters.

Alterations in the immune-related gene expression of the coinfected fish were assessed by analysing the levels of the cytokines *ifn-γ* and *il-8*, and the antiviral genes *mx* and *rsad2* in the anterior kidneys of the coinfected fish (Figure 5). Specifically, the expression levels of *ifn-γ* were comparable across all the groups at 3 dpi, with the average relative expression ranging from 0.37- to 1.1-fold (Figure 5A). At 5 dpi, the expression of *ifn-γ* in the fish that were fed additive with strategy A increased markedly to 7.7 ± 5.5-fold, which was significantly higher than that in the other groups (*p* < 0.05). However, the levels of *ifn-γ* in these fish declined gradually and showed no significant differences across any of the groups at 7 dpi (1.1- to 2.8-fold) and 14 dpi (0.23- to 0.56-fold).

The expression levels of *il-8* were remarkably altered in the fish that were fed the control and strategy A additives (Figure 5B). By 3 dpi, the *il-8* levels had increased 9.8 ± 3.3-fold in the control group and 14 ± 4.2-fold in the strategy A group, and these increases were significantly higher than those in the fish that were fed strategy B additives (*p* < 0.05). While the *il-8* levels remain unchanged at 5 dpi across all the groups, a significant reduction was observed at 7 and 14 dpi. Notably, at 7 dpi, the fish that were fed with strategy A showed *il-8* levels of 4.8 ± 3.3-fold, which were significantly higher than those in the fish that were fed other diets (*p* < 0.05). By 14 dpi, the levels of *il-8* had decreased across all the groups, with no significant differences between them.

Conversely, the expression of the antiviral genes *mx* and *rsad2* showed minor changes throughout the study period (Figure 5C,D). Specifically, at 3 dpi, the *mx* expression in the fish that were supplemented with strategy B was 4.2 ± 1.9-fold, which was lower than that in the control group (*p* < 0.05) (Figure 5C). Notably, the expression of both genes remained relatively consistent across all groups at 5 dpi. By 7 dpi, the *mx* expression had reduced significantly in all the groups, with the control group maintaining the highest level at 1.9 ± 0.9-fold (Figure 5C). The level of *mx* in the fish that received strategy B was 0.19 ± 0.08-fold, which was significantly lower than that in the control group (*p* < 0.05). Likewise, the *rsad2* expression in the fish fed the control diet increased markedly to 4.2 ± 2.4-fold at 7 dpi, which was significantly higher than in the supplement diet groups (*p* < 0.05) (Figure 5D), while the fish that were fed strategy B additives showed the lowest expression of *rsad2* at 0.31 ± 0.26-fold at 7 dpi. By 14 dpi, the expression levels of both anti-viral genes had decreased and were comparable across all the groups.

### 3.4. Histopathological Changes

**Figure 6:**
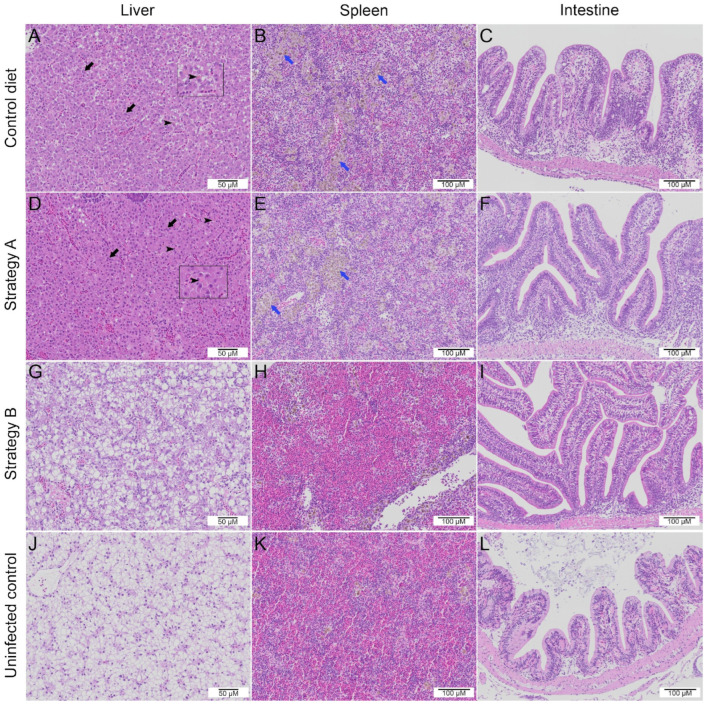
Histopathological changes at 7 days post-infection in the liver, spleen, and intestines of red hybrid tilapia coinfected with Tilapia lake virus and *Aeromonas hydrophila*: comparison between the groups that received the control diet (**A**–**C**) or additive-supplemented feed (**D**–**I**) and the uninfected control group (**J**–**L**). Syncytial hepatocytes (black arrows), the presence of intracytoplasmic inclusion bodies in the liver (arrowheads, inset), the severe depletion of red blood cells, and the distinct proliferation of melanomacrophage centres in the spleen (blue arrows) are denoted.

We examined the histopathological changes in the internal organs, including the liver, spleen, and intestines, of all the challenged fish at different time points (Figure 6, Supplementary Figures S2 and S3). Specifically, the infected fish that were fed the control diet exhibited remarkable histopathological alterations at 5 and 7 dpi. These changes included syncytial cell formation, glycogen storage depletion, and the presence of intracytoplasmic inclusion bodies in the liver; the severe depletion of red blood cells and the distinct proliferation of melanomacrophage centres (MMCs) in the spleen; and lymphocytic infiltration concurrent with widening of the lamina propria and the loosening of enterocytes from the basal membrane in the intestines (Figure 6A–C, Supplementary Figure S2). Interestingly, the fish supplemented with strategy B showed fewer pathological changes, such as mild syncytial hepatitis in the liver, abundant red blood cells in the spleen, and a mild increase in intestinal goblet cell numbers (Figure 6G–I) compared to the infected control group. In addition, the fish that were fed with strategy A had a slight increase in goblet cells in the intestines, while the liver and spleen showed severe pathological changes (Figure 6D,E) that were similar to those in the coinfected control group. At 14 dpi, hepatic lesions persisted in the livers of the fish in both the control group and the strategy A-supplemented group, whereas no hepatic lesions were observed in the additive B group (Supplementary Figure S3). Lastly, all the groups, except the strategy B-supplemented group, showed an increase in MMCs in the spleen, but no distinct pathological changes were observed in the intestines of any of the groups at 14 dpi.

## 4. Discussion

Coinfections of bacteria and viruses, including those involving TiLV, are commonly reported in tilapia [5,7]. Although it is challenging to determine whether viral or bacterial infections occur first in natural aquaculture settings, previous observations have shown that TiLV often leads to severe disease and is associated with remarkable pathological changes [5,7]. Therefore, in this study, we introduced TiLV infection prior to the bacterial challenge. Bacteria in the genera Aeromonas, Streptococcus, and Edwardsiella are often isolated from fish infected with TiLV, and these pathogens lead to severe clinical outcomes and high mortality rates [19,20]. This situation highlights the need for effective strategies to mitigate the impacts on tilapia aquaculture. Currently, no specific antiviral treatment is available for TiLV. To avoid the overuse of antibiotics and to tackle the challenge of coinfections with TiLV and bacteria, alternatives, such as probiotics and feed additives that modulate the immune system and enhance resistance to bacterial infections, offer promising options. In this study, tilapias were fed diets supplemented with two feed additives and subsequently challenged with TiLV and *A. hydrophila*. Our findings demonstrated that coinfection with TiLV and *A. hydrophila* resulted in high mortality, severe clinical signs, and the increased expression of proinflammatory and immune-related genes. These results are consistent with those of previous studies, which have shown that TiLV alone or in combination with bacteria can cause significant mortality in various species of tilapia under laboratory conditions [21,22] and in natural environmental settings [23].

Feed additives have become essential components in tilapia diets to promote growth performance, boost immunity, balance gut pH and microbiota, and prevent tissue damage from virulent pathogens [11,24,25]. Our study demonstrated the beneficial effects of feed additives with immunomodulatory properties in red hybrid tilapia. Specifically, strategies A and B, which contained blends of short- and medium-chain fatty acids, significantly reduced the mortality of the tilapia coinfected with TiLV and *A. hydrophila*. Previous studies have shown that these organic acid blends exhibit antimicrobial activity against *A. hydrophila* and *Francisella orientalis* and can have positive effects that reduce mortality in tilapia [24]. Furthermore, commercial phytogenic feed additives with immunomodulatory properties have been reported to improve the survival of Atlantic salmon when challenged with *Piscirickettsia salmonis* [26]. Indeed, some feed additives have been shown to reduce the viral load [27,28] and decrease the bacterial burden in the tissues of infected fish [29]. Although the levels of TiLV and *A. hydrophila* detected in the tissues of the experimental fish in this study did not differ significantly between the two groups that received the feed additives, the fish supplemented with short- and medium-chain organic acids showed better survival rates than those that were fed a regular diet. It therefore appears that these organic acids may mitigate the pathological effects of infection through immunomodulation of the host rather than direct antiviral or antimicrobial action.

The reduced mortality in the groups supplemented with strategies A and B in our study could be attributable to the differences in the expression of the genes related to inflammation and pathogen control. Notably, the group fed additive B had lower mortality, fewer pathological changes, and a lower expression of the immune-related genes *il-8*, *mx*, and *rsad2. Il-8*, which is a member of the CXC chemokine family, plays a crucial role in recruiting immune cells to infection sites and modulating their function [30]. It serves as a key player in innate immunity and inflammatory responses against pathogens [31]. Similarly, the *mx* and *rsad2* genes are important regulators in the innate immune response of fish against viral infections, as they inhibit early viral replication and disrupt viral assembly and budding [32]. Our observation revealed an increased expression of *il-8*, *mx*, and *rsad2* transcripts in TiLV-infected fish, which is consistent with findings from previous studies [17,33]. In the strategy B supplemented group, the decreased expression of *il-8* at 3 dpi suggests a reduction in early inflammatory responses, which may limit tissue damage. Notably, the lower expression of *mx* and *rsad2* at 7 dpi, compared to the control group, highlights the immunostimulatory effects of this additive. This finding suggests that strategy B may promote initial antiviral defences, as seen in the early expression of *mx*, thereby enabling the fish to manage the viral infection more effectively, reducing the need for prolonged activation of these genes [33]. During TiLV infection, the intestinal epithelium is a primary entry point for TiLV pathogenesis [34,35]. Since both TiLV and *A. hydrophila* infection can disrupt the gut microbiota in the intestines of tilapia [36,37], we hypothesised that acidifiers—the organic and amino acids in strategy B—alleviate pathological outcomes during coinfection with these pathogens. These acids could reduce intestinal inflammation, modulate gut microbiota, and boost gut immunity, supported by similar findings in tilapia infected with *F. orientalis* and shrimp exposed to *Vibrio parahaemolyticus* [24,38,39]. Hence, supplementation with additives following strategy B may promote the activities of the immune system and improve the gut health of tilapia during coinfection, thus leading to higher survival rates.

Notwithstanding, the coinfected tilapia supplemented with strategy A, which consisted of lyso-phospholipids, showed lower mortality rates but displayed severe pathological lesions in the liver and spleen. The fish treated with strategy A showed an increased expression of the proinflammatory cytokine *il-8* and decreased expression of *rsad2* at 7 dpi, which suggested that additive A did not provide protective effects against inflammation induced by the coinfection, with significant tissue damage as a potential outcome. However, we found fewer intestinal abnormalities in the fish fed with strategy A compared to those that were fed a regular diet. Previously, lyso-phospholipids have been shown to improve enterocyte mucous production and cellular responses against viral infection and stress in Atlantic salmon [40]. Lyso-phospholipids can promote nutrient metabolism in hepatocytes and enterocytes [41], which may contribute to the increased survival of coinfected tilapia. We hypothesised that the efficacy of supplementation using strategy A in improving fish survival is attributable to its role in intestinal protection, which includes the better absorption and utilisation of nutrients in the intestines and liver. This may help improve the health status of the tissue and reduce the impact of disease. Nevertheless, future investigations into the intestinal molecular bioenergetics, blood chemistry, and other panels of immune-related genes in the coinfected fish that receive these additives are necessary to fully understand the mechanisms by which they protect against coinfections. Additionally, factors such as water quality and other concurrent infections in farm settings can significantly influence the immune system and responses to dietary supplementations. Therefore, when selecting strategies to prevent diseases in tilapia, these factors should be taken into account. Overall, although the supplementation of each feed additive showed positive results under laboratory conditions, further field studies should be conducted to strengthen the evidence of the beneficial effects of these additives against coinfection in tilapia.

## 5. Conclusions

In our study, we demonstrated that dietary supplementation with specific feed additives can mitigate the negative impacts of TiLV and *A. hydrophila* coinfection in tilapia. Specifically, strategy B, which contained organic acids and natural immunostimulants, effectively reduced mortality and modulated the immune response of the fish, while strategy A, which contained organic acids and lyso-phospholipids, had a significantly protective effect on the fish intestines. Further investigations and field studies are necessary to fully understand and optimise the use of these additives to combat TiLV and *A. hydrophila* coinfection in tilapia farms.

## Figures and Tables

**Table 1:** Details of the primer pairs used for the quantitative polymerase chain reaction.

Gene Name	Accession Number	Primer Sequence 5′ → 3′	Length (bp)	References
TiLV	KU751816	F: CTGAGCTAAAGAGGCAATATGGATT	112	[15]
		R: CGTGCGTACTCGTTCAGTATAAGTTCT		
*β-actin*	XM003443127	F: GTGGGTATGGGTCAGAAAGAC	111	[17]
		R: GTCATCCCAGTTGGTCACAATA		
*il-8*	NM001279704	F: TCGCCACCTGTGAAGGCA	116	[11]
		R: GCAGTGGGAGTTGGGAAGAAT		
*ifn-γ*	NM001287402	F: GAAACTTCTGCAGGGATTGG	132	[18]
		R: CTCTGGATCTTGATTTCGGG		
*mx*	XM003442686	F: ACCCTTGAGCTGGTGAATCA	174	[18]
		R: ATCCTGAGTGAATGCGGTCA		
*rsad2*	XM003453237	F: ATCAACTTCTCTGGCGGA	161	[11]
		R: AGATAGACACCATATTTCTGGAAC		

**Table 2:** Number of fish in which *Aeromonas hydrophila* was detected from the anterior kidney of the fish challenged with Tilapia lake virus (TiLV) and *A. hydrophila*.

	**dpi ^†^**	**Positive Samples/Total Samples**				
**Groups**		0	3	5	7	14
Sham		0/3	0/3	0/3	0/3	0/3
Control diet		0/3	0/3	3/3	3/3	0/3
Strategy A		0/3	0/3	3/3	3/3	0/3
Strategy B		0/3	0/3	3/3	3/3	0/3

**^†^** dpi, days post-infection.

## Data Availability

The data presented in this study are available on request from the corresponding author. The data are not publicly available due to the anonymity granted to all participating parties.

## Supplementary Materials

The following supporting information can be downloaded at https://www.mdpi.com/article/10.3390/biology13110938/s1, Figure S1: Detection of *Aeromonas hydrophila* in the anterior kidney of the coinfected fish at 7 days post-infection. (A) Representative agarose gel showing the polymerase chain reaction product (130 bp) of the *GyrB* gene specific against *A. hydrophila*. (B) Representative figures showing bacterial growth on Tryptic soy agar isolated from the anterior kidneys of infected tilapia in the control and strategy A and B groups; Figure S2: Histopathological changes at 5 days post-infection in the liver, spleen, and intestines of red hybrid tilapia coinfected with Tilapia lake virus and *Aeromonas hydrophila* that received the control diet or additive-supplemented feed. Syncytial hepatocytes (black arrows), the presence of intracytoplasmic inclusion bodies in the liver (arrowheads, inset), the severe depletion of red blood cells, and the distinct proliferation of melanomacrophage centres in the spleen (blue arrows) are denoted; Figure S3: Histopathological changes at 14 days post-infection in the liver, spleen, and intestines of red hybrid tilapia coinfected with Tilapia lake virus and *Aeromonas hydrophila* that received the control diet or additive-supplemented feed. Supplementary Table S1. Nutrient compositions of control, strategy A and B feed.
