# Supplementary material for: Efficacy of Feed Additives on Immune Modulation and Disease Resistance in Tilapia in Coinfection Model with Tilapia Lake Virus and Aeromonas hydrophila
Source: Biology (Basel). 2024 Nov 16;13(11):938. doi: 10.3390/biology13110938 (PMC11591586; doi:10.3390/biology13110938)
Supplement: Supplementary file 1 [file biology-13-00938-s001.zip › biology-3290589-supplementary.pdf]

Supplementary Materials:

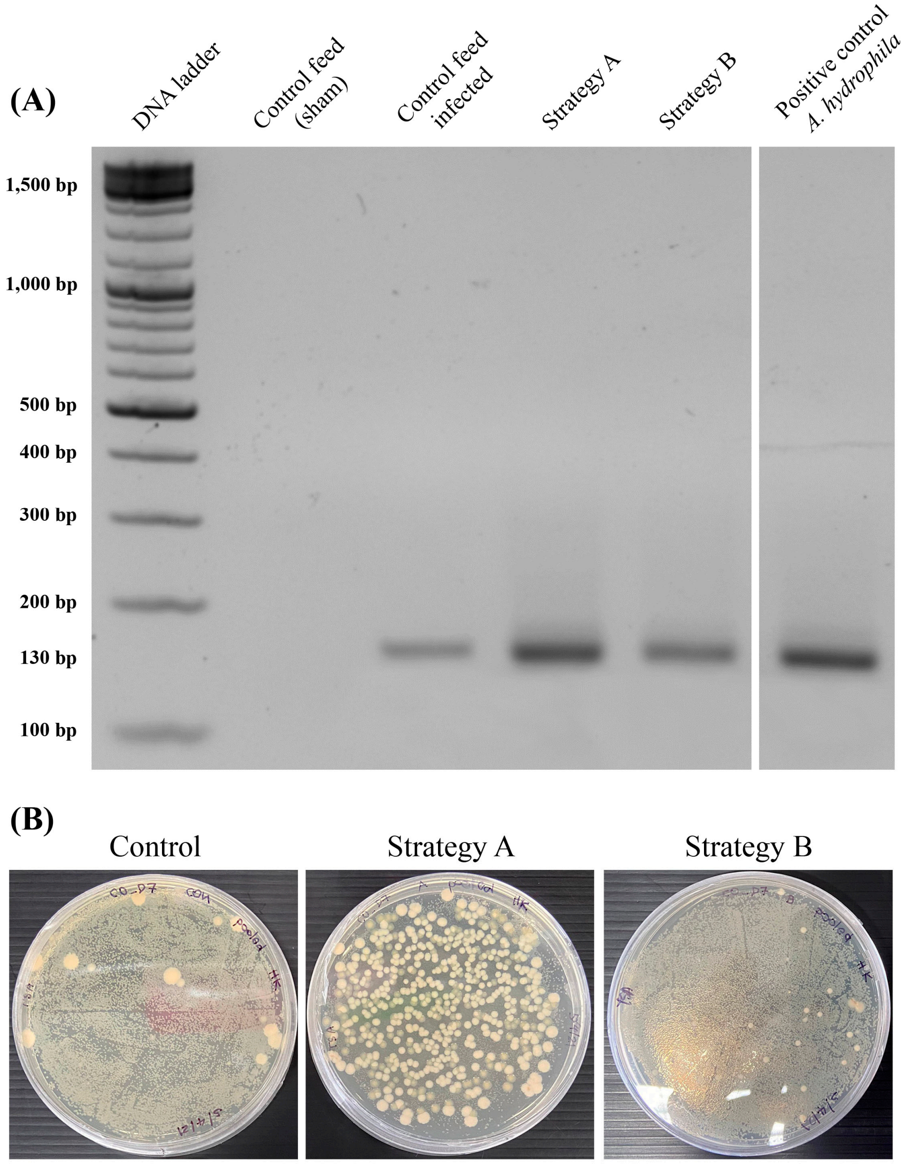

Figure S1: Detection of *Aeromonas hydrophila* in the anterior kidney of the coinfected fish at 7 days post-infection. (A) Representative agarose gel showing the polymerase chain reaction product (130 bp) of the GyrB gene specific against *A. hydrophila*. (B) Representative figures showing bacterial growth on Tryptic soy agar isolated from the anterior kidneys of infected tilapia in the control and strategy A and B groups.

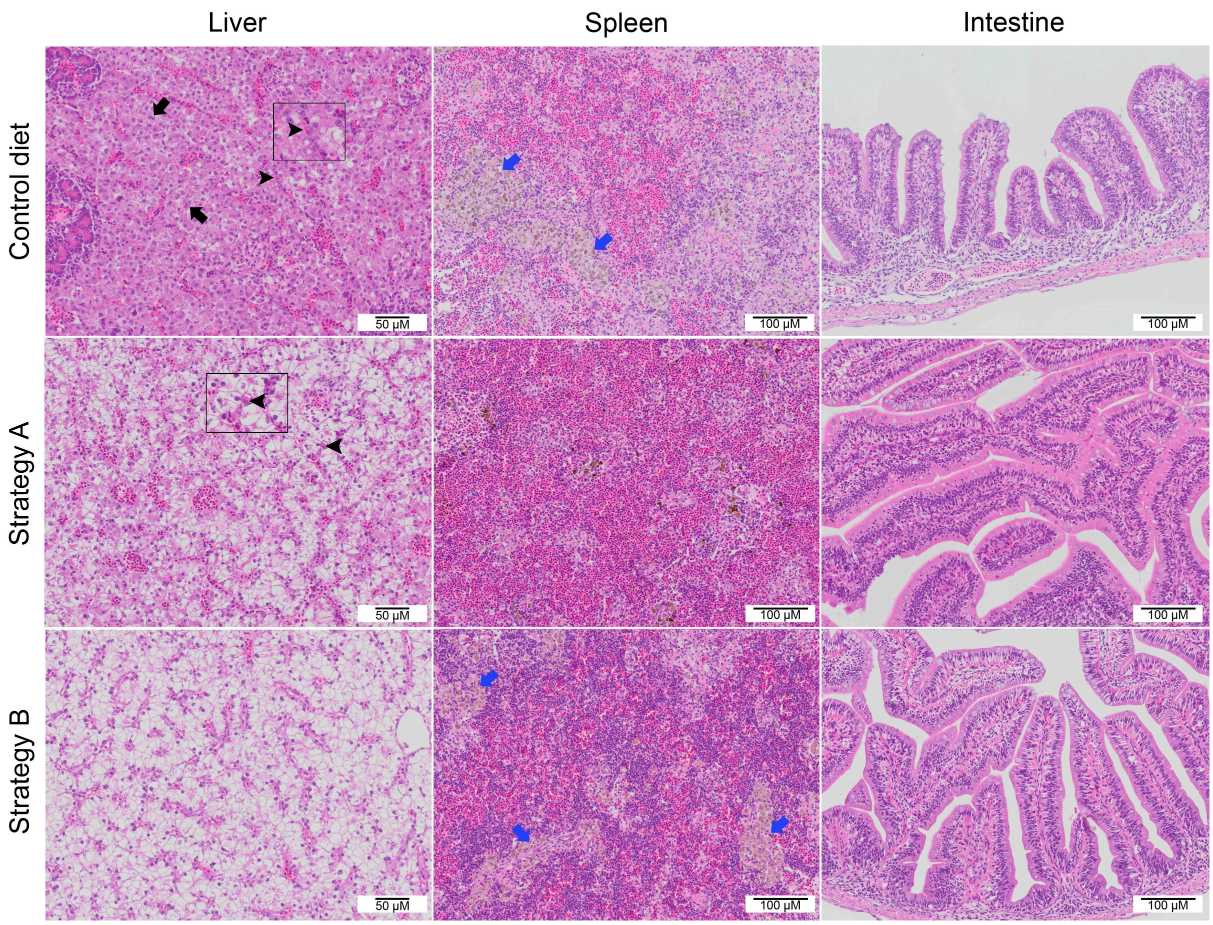

Figure S2. Histopathological changes at 5 days post-infection in the liver, spleen and intestines of red hybrid tilapia coinfected with Tilapia lake virus and *Aeromonas hydrophila* that received the control diet or additive-supplemented feed. Syncytial hepatocytes (black arrows), the presence of intracytoplasmic inclusion bodies in the liver (arrowheads, inset), the severe depletion of red blood cells and the distinct proliferation of melanomacrophage centres in the spleen (blue arrows) are denoted.

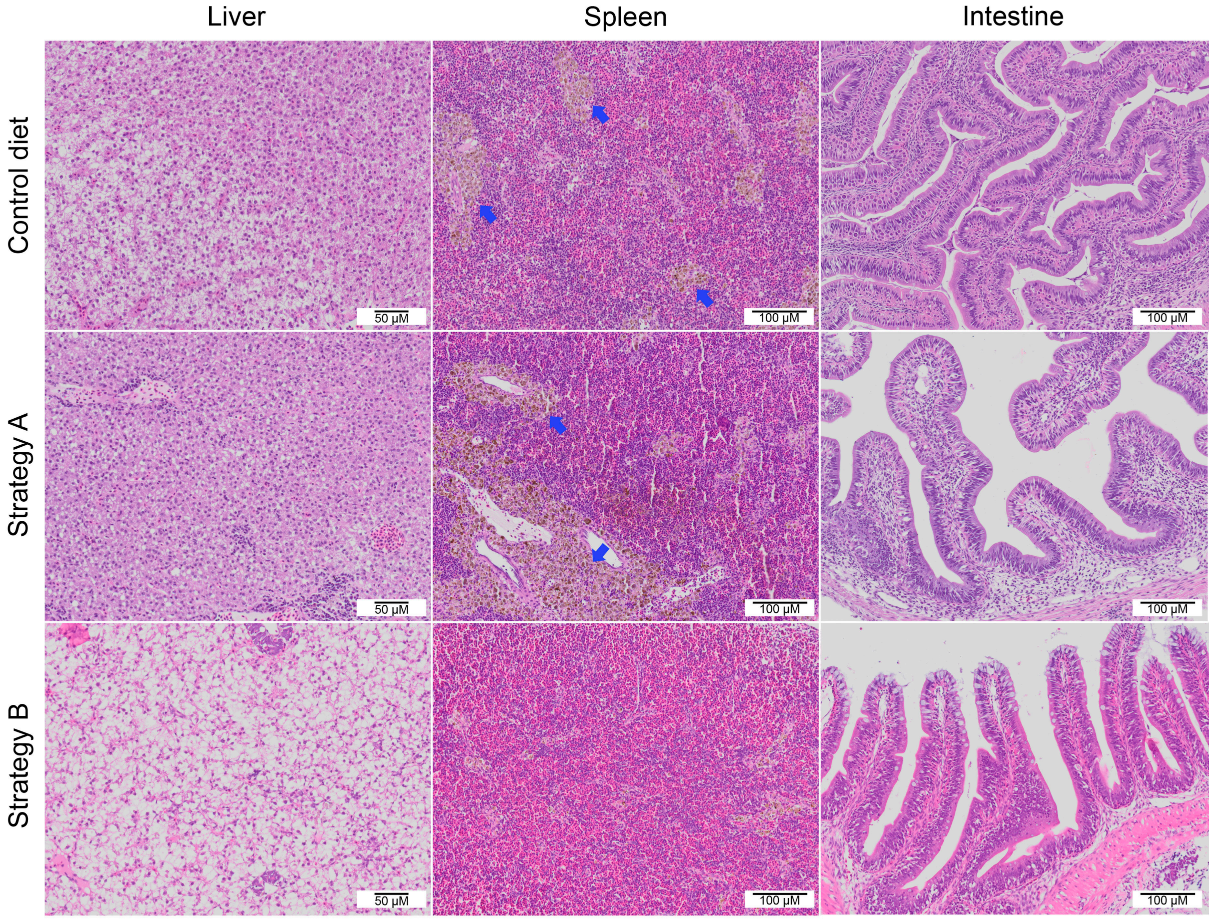

Figure S3. Histopathological changes at 14 days post-infection in the liver, spleen and intestines of red hybrid tilapia coinfected with Tilapia lake virus and *Aeromonas hydrophila* that received the control diet or additive-supplemented feed.

Supplementary Table S1. Nutrient compositions of control, strategy A and B feed.

**Nutrient composition**	**Percent (% dry matter)**
Fat	10.00
Crude protein	32.90
Arginine	2.08
Lysine	2.10
Methionine + cysteine	1.13
Threonine	1.27
Tryptophan	0.37
Total calcium	1.91
Available phosphorus	0.70
Strategy A	0.3% Bacti-Nil Aqua + 0.1% Aqualyso + 0.1% wheat
Strategy B	0.3% Bacti-Nil Aqua + 0.1% Aquastim + 0.1% wheat
Control	0.5% wheat
